# Probing Stochastic Nano-Scale Inelastic Events in Stressed Amorphous Metal

**DOI:** 10.1038/srep06699

**Published:** 2014-10-21

**Authors:** Y. Yang, X. L. Fu, S. Wang, Z. Y. Liu, Y. F. Ye, B. A. Sun, C. T. Liu

**Affiliations:** 1 Centre for advanced structural materials, Department of Mechanical and Biomedical Engineering, City University of Hong Kong, Tat Chee Avenue, Kowloon TongKowloon, Hong Kong, P.R. China

## Abstract

One fundamental yet longstanding issue in materials science is how local inelasticity arises within an amorphous structure before yielding occurs. Although many possible scenarios were postulated or predicted by theories and simulations,however, direct experimental evidence has been lacking today due to the lack of a sensitive way to detect nano-scale inelasticity. Through the carefully designed microcompression method as coupled with the state-of-art nano-scale electric resistance measurement, we here unfold a stochastic inelastic deformation process in a Zr-based metallic glass, which takes place via the recurrence of two types of short-lived inelastic events causing structural damage and recovery, respectively, prior to yielding. Our current findings reveal that these stochastic events not only self-organize into sub-critical events due to elastic coupling, but also compete with each other in a way that enables the whole amorphous structure to self-heal as well as to sustain local damage.

Amorphous materials are ubiquitous in our everyday life^1^. Window glass, bio-polymers, gels, and thin-film semiconductors are the typical examples of amorphous material. Despite their different chemical compositions, the basic structures of these amorphous materials are similar, composed of atoms, molecules and grains arranged in a ‘disordered' manner resembling that of liquids. However, unlike liquids flowing indefinitely under an external stress, amorphous solids exhibit a yield-stress behavior similar to their crystalline counterparts at a finite time scale, even though the structure of the amorphous solids possesses no long-range translational symmetry and thus no dislocation-like defects to initiate a plastic flow.

Over the past decades, it is the long-sought issue with regard to the origin of flow in amorphous solids that has been attracting tremendous research interest of materials scientists and physicists^2^,^3^,^4^,^5^,^6^,^7^,^8^,^9^,^10^,^11^,^12^,^13^,^14^,^15^,^16^. As any structural changes occurring before yielding in amorphous solids are presumably 'tiny' and short-lived, conventional mechanical tests alone cannot be used to detect them. Despite that a great number of atomistic simulations were conducted^3^,^4^,^8^,^10^,^11^,^14^, providing important insights into the atomic or molecular scale mechanisms, however, they were usually carried out under extreme conditions, such as the unrealistically high deformation rate, low temperature and small sample size, which can hardly be verified experimentally. Therefore, as of today, this problem still remains in an intense debate due to the lack of the key experimental evidence which is able to underpin the mechanisms of flow initiation in amorphous solids. This disconnection between experiments and theories/simulations limits the scientific understanding and thus, practical applications of the variety of amorphous solids.

Here, a coupled nano-scale electro-mechanical system was used to tackle this problem. As seen in the later text, this system is proven to be extremely sensitive in resolving the structural signals that result from inelastic events occurring during the apparent 'elastic' deformation of a metallic glass. As compared to a conventional mechanical test, the nano-mechanical module of this system is based on the recently developed Hysitron TI950 nanoindentation platform, which possesses the resolution of ~0.1 nm in displacement and ~1 μN in force; in addition, the coupled nano-electric module is based on the state-of-art Nano-Electric-Contact-Resistance (NanoECR) technique, which possesses the resolution of 10–100 pA in current and 0.1 mA in voltage [See Fig. S1 in the Supplementary Information (SI)]. The coupling of these two modules enables us to track any 'tiny' structural change that would occur within a metallic glass under stress, as shown in Fig. 1(a).

For this study, the model material we used is a Zr-based metallic glass with the chemical composition of Zr_55_Cu_30_Ni_5_Al_10_ (in at. %). Its structural amorphousness was confirmed using the X-ray diffraction method and high-resolution transmission electron microscopy (See Figs. S2–S3 in SI). To simplify the data collection and analysis, the microcompression method was used to deform the metallic glass. Multiple metallic-glass micropillars were fabricated using the focused ion beam (FIB) technique^17^, which had a diameter of ~2 μm and an aspect ratio of ~2.5. Microcompression was conducted on the Hysitron TI950 nanoindentation platform at load control. A load function, consisting of loading, holding and unloading segments, was applied, onto which a constant voltage of 5 V was superimposed to extract the electric properties of the metallic glass in situ [Fig. 1(b)].

Figure 1(c) shows the typical displacement (*h*) and electric current (*I*) data, as a function of time (*t*), measured simultaneously from the microcompression test. It should be emphasized here that, due to the surface roughness effect and the settling of the nanoindenter on the micropillar, there is a period of 1–2 seconds before an electric Ohm's contact could be established (See ‘*Electric Contact Analysis in Microcompression*' in the SI). Therefore, for the sake of simplicity, only the data collected after the settling time were chosen for the data analyses. After considering both the geometric and size change induced by the samll deformation in the tapered micropillar, a simple relation can be derived 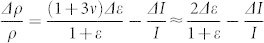 (see ‘*Ohm's Law in Microcompression*' in the SI), which relates the electric resistivity *ρ* of the metallic glass to the measured nominal or engineering strain *ε*, the Poisson's ratio ν(≈1/3) and the electric current *I*. In the above relation, *Δ* represents an incremental change of a physical quantity and *ε* = *h/H_0_*, where *H_0_* denotes the initial height of a micropillar. Here it should be noted that, due to the ultrahigh sensitivity of our testing system, the noise-to-signal ratio for the calculated *Δρ/ρ* is very low and about 1 × 10^−4^ (see '*Error Analysis*' in the SI).

With the above equation, the relative change in the electric resistivity (*Δρ/ρ*) of the metallic glass can be extracted from the measured displacement (*h*) and current (*I*) data. Figure 2(a) shows the typical curves of *Δρ/ρ* and *h* versus time *t* as the micropillar was compressed to a peak stress of ~2000 MPa. Before reaching this peak stress, a few pop-in events can be witnessed on the *h-t* curve. Note that, for the sake of consistency, a displacement pop-in event is herein defined as a displacement jump greater than ~2 nm in size. In accord with the pop-in events, a few pronounced resistivity spikes can be also observed on the *Δρ/ρ* -*t* curve. As seen in the inset of Fig. 2(a), these resistivity spikes, ranging from 0.001 to 0.1 in magnitude, correlate very well with the pop-in strains. As pop-in events originate from shear banding in metallic glasses^4^,^5^,^6^, therefore, the resistivity spike corresponding to the electric resistivity increase signifies a deformation-induced 'damage' in the metallic glass. Most interestingly, besides these pronounced resistivity spikes occurring concomitantly with displacement pop-in, many secondary spikes also appear before or after a typical pop-in event. As shown in Figs. 2(b)–(d), parts of these secondary spikes point upwards (p-type spikes), indicating the electric resistivity increase in the metallic glass like those at displacement pop-in; while parts of them point downwards (n-type spikes), indicative of the electric resistivity reduction. Note that the smallest secondary spikes possess a magnitude |*Δρ/ρ*| greater than 5 × 10^−4^, which is about fivefold of the maximum experimental error that could occur to the derived resistivity change. As such, we believe that these secondary spikes are the manifestation of real structural alteration caused by the local inelastic events in the metallic glass.

Physically, the resistivity, *ρ*, of a metallic glass can be related to its amorphous structure through the Ziman's model^18^, which is: 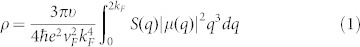
where *υ* is the atomic volume, *e* the electric charge of a 'free' electron, *k_F_* the Fermi wave vector, *v_F_* the Fermi velocity, *S(q)* the structure factor, and μ(q) the pseudo-potential which can be replaced by a scattering matrix when the above model is applied to metallic glasses. In the 1980s, Kelton and Spaepen^19^ showed that, upon external stimulation, the resistivity change, *Δρ*, of a metallic glass can be directly related to the variation in its atomic packing density *Δξ* through *Δρ = CΔξ*. Here *C* is the pre-factor that depends mainly on the structural factor *S*(*q*) of the metallic glass (See ‘*Ziman's Model*' in the SI). Since the change in the structural factor of metallic glasses is insignificant during elastic deformation and thermal annealing^19^, we here assume *C* to be a constant. In such a case, the electricity fluctuation as we measured can be solely ascribed to the density fluctuation. In what follows, let us discuss the possible relationship between the electricity and density of metallic glasses. According to Mooij^19^,^20^, the change in the electric resistance *Δρ* of a metallic glass is correlated with the change in its density under thermal annealing. However, this correlation depends on the initial thermal expansion coefficient α of the metallic glass. In general, it was found that electricity decreases (*Δρ* < 0) with thermal annealing if *α* < 5 × 10^−5^ K^−1^ or otherwise increases with thermal annealing. On the other hand, based on the data from 13 types of metallic glasses with different glass transition temperatures *T_g_*, Kato and co-workers^21^ discovered that *αΤ_g_* ~ 8.24 × 10^−3^ for metallic glasses. As the *T_g_* of Zr-based metallic glasses are around 700 K, the corresponding *α* should be about 1.2 × 10^−5^ K^−1^. This suggests that the electric resistance of Zr-based metallic glasses should decrease with thermal annealing or structural ordering, which is consistent with the previous result reported by Bai and co-workers^22^. Along this line of reasoning, we can therefore ascribe the increase in *Δρ/ρ* of the Zr-based metallic glass to structural disordering while the decrease to structural ordering. In other words, the recurrence of these p- and n-type spikes indicates that the atomic structure of the metallic-glass micropillar was repeatedly experiencing damage and recovery even though the applied stress was far below the yielding point.

Figures 2(b) to (d) display respectively the magnified views of the secondary resistivity spikes arising during the ‘elastic' loading, holding and unloading segments. As seen in these figures, the measured displacement *h* increases with time *t* during loading [Fig. 2(b)], levels off to a constant during holding [Fig. 2(c)] and decreases during unloading [Fig. 2(d)]. Note that, within the data scattering < 1 nm, there are no observable pop-in events on the *h-t* curves, agreeing with the ‘elastic' character of the overall deformation in the metallic glass. In sharp contrast, the corresponding curves of *Δρ/ρ* versus *t* display many spike-like features. Compared to those prominent spikes at displacement pop-in, these spikes occurring in the ‘elastic' regime appear much shorter and last only for a short period of time. Due to the limit of our data acquisition rate (~10^3^ points per second), the measured duration times *Δt_d_* for these secondary spikes range from ~1 to ~1.5 ms and appear to be independent of the spike heights and the applied stresses (See *‘Spike Lasting Time'* in the SI). By comparison, a pop-in event generally lasts much longer, the duration of which scales with the pop-in strain and can reach up to ~8 ms. This behavior suggests that, unlike the pop-in or shear banding events which are size dependent^4^,^5^, the local inelastic events behind the secondary spikes behave in a stochastic manner, which may be scale independent.

To further characterize the stochasticity of these secondary spikes, we performed a statistical analysis to calculate the probability for the occurrence of the secondary spikes. For this purpose, the total number of the secondary spikes with the same magnitude |*Δρ/ρ*| is added up irrespective of the spike sign and the timing when the spike is spotted. The inset of Fig. 3(a) displays histogram of these secondary spikes, which was obtained from the microcompression test with the peak stress of 740 MPa. Interestingly, the statistical distributions of these secondary spikes exhibit the similar trend regardless of the peak stress [Fig. 3(a)]. Over the range of the spike magnitude from 10^−4^ to 10^−2^, these distributions can be fitted very well to the empirical relation 
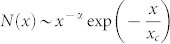
 where *α* is the power-law exponent characterizing the stochastic behavior of the spikes having a magnitude < *x_c_*, in which *x_c_* is a critical magnitude above which the spike frequency exhibits exponential decaying, as shown in Fig. 3. Through the nonlinear data fitting, α is obtained to be 1.5 ± 0.1 (See *‘Estimation of Power Law Exponent'* in the SI) with *x_c_* ~ 10^−3^.

Now let us discuss the underpinning physics behind our experimental observations. In the metallic-glass literature, it is a longstanding notion that metallic glasses are dynamically heterogeneous, which can be viewed as a ‘mixture' composed of loosely- (liquid-like) and densely-packed (solid-like) atoms^8^,^9^,^10^,^23^,^24^,^25^,^26^,^27^,^28^, as illustrated in Fig. 3(b) or (c). Upon mechanical loading, inelastic events, such as shear transformation events, may occur in the regions centered in these liquid-like atoms, the behavior of which resembles viscous ‘flow units'^29^,^30^,^31^ accommodating inelastic deformation through local configurational rearrangements, whilst the solid-like ones behave elastically upholding the integrity of the amorphous structure. Within the framework of the classic shear-transformation-zone (STZ) model^32^, it was postulated that these inelasticity events could only lead to structural disordering. However, our current results clearly indicate that structural disordering is only one possible outcome of these inelastic events while the other possible outcome is structural ordering or densification. Furthermore, our results also show that the occurrence of these inelastic results is stochastic, which supports the assumption of stochastic shear transformation as we recently proposed in developing a mean-field anelastic model for MGs^29^,^33^,^34^.

Assuming a probability *p*(*τ,t*) for the net transition of local structural configurations along the stress direction at time *t* and shear stress *τ*, it can be proved that *p*(*τ,t*) satisfies the following dynamic equation in order to retain the total free energy of the system^29^,^33^: 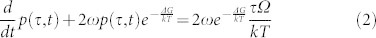
where *ω* is the attempt frequency; *ΔG* is the energy barrier and *Ω* is the activation volume of the local inelastic event. For a constant shear stress τ at load hold, we obtain: 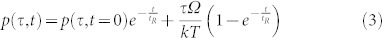
where *t_R_* is the average relaxation time of these inelastic events, which equals 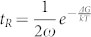. From Eq. (3), it can be inferred that, when *t/t_R_* goes to infinity, the ‘memory' of the MG about the stochastic inelastic events at *t* = 0 will die out while the probably *p*(τ,t) for the net transition along the stress direction will approach a steady-state value *p*(τ) = *τΩ*/*kT*. Note that Eq.(2) was derived for the simple case of a constant activation volume. This simplification can be eliminated by taking into account the possible distribution of the activation volume in a metallic glass. Thus, the real probability for the net configurational transition at the given size *Ω* should be *p*(*τ*)*f*(*Ω*)d*Ω*, where *f* is the probability density function of the activation volume for an inelastic event. If the ‘liquid-like cell' model of Cohen and Grest^23^ is followed, it can be shown that 
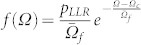
, where *p_LLR_* is the volume fraction of the liquid-like cells, 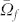 the activation volume averaged over all liquid-like cells while *Ω_c_* as the smallest activation volume. In such a case, the following cumulative probability can be obtained for the constant stress activation of the local inelastic events with their activation volumes greater than *Ω*: 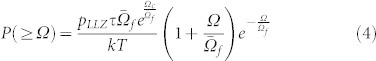


With Eq. (4), we consider a general case that each spike event as detected from the micropillar involves a series of different sized activations. As such, the probability for these spatially isolated activation events decay exponentially when the smallest activation size involved grows, as indicated by Fig. 4(a).

To verify the above thinking, let us follow the recent analysis of Krisponeit et al.^35^, in which the total activation volume or slip as involved in an inelastic deformation event is correlated with the incubation or waiting time *Δt_int_* ahead of the event. Experimentally, *Δt_int_* can be measured as the time interval between two consecutive spike events of the same kind. Figures 4 (b)–(c) display the histograms of *Δt_int_* obtained for the holding stresses of 296 MPa and 592 MPa, respectively. Consistent with our theory, the general trend of the waiting times *Δt_int_* clearly shows the distribution of an exponential decay. Meanwhile, as the holding stress is doubled, it appears that longer waiting times could be detected, which also agrees with our prediction. However, it needs to point out that this agreement is only in a qualitative manner. Rather than the strong linear stress dependence, the stress dependence of the accumulative probability, as seen from our data, is very weak. A similar behavior could be observed at other holding stresses. This weak stress dependence suggests that the simple mean-field anelastic model we proposed needs further development. One possible way of improvement is to include a term of elastic coupling into the mean-field modeling. In such a case, we can study how the variation of the stress field is coupled with the variation of the local properties, which may explain the weak stress dependence shown by our experimental data. The related theoretical work is under progress and will be discussed in our future work. Despite the weakness of the mean-field modeling, it is still worth mentioning that, with the mean-field model and *Δt_d_*~ 1 ms, we can estimate an average activation energy of ~0.5 eV for these local inelastic events, which agrees with the previous results obtained from nanoindentation^29^,^34^ (See ‘*The Estimation of Activation Energy*' in the SI).

Next, we would like to further address the following questions: in what cooperative way these chain-like inelastic events occur? And even if the occurrence of these events is stochastic in nature, are their outcomes, such as the structural relaxation and damage, also stochastic? In principle, these local inelastic events may occur in an isolated manner as pictured in the previous mean-field theories^15^,^32^, or cooperatively through different mechanisms. As illustrated in Fig. 3(b), one possible mechanism of the cooperative activation is through elastic coupling: as one inelastic event takes place, it alters the elastic stress field nearly, thus triggering another inelastic event and so on and so forth, until eventually an avalanche event occurs; alternatively, the other possible mechanism involves de-caging or break-down of a local elastic environment, which leads to the coalescence of the neighboring liquid-like regions [Fig. 3(c)]. In the literature, theoretical models have been developed to account for the former case of the cooperative motion^7^. According to Dahmen et al.^7^, should elastic coupling govern the cooperative motion, the distribution of the avalanche magnitude is of a power law with the exponent of ~1.5^7^. Comparing our experimental result with this theoretical prediction, it appears that the secondary spikes, particularly for those with a small magnitude (<10^−3^), is likely to be caused by the avalanches triggered through elastic coupling. Nevertheless, we envision that the latter mechanism due to the de-caging effect is also feasible for the high-magnitude spikes; however, the probability for its occurrence must be very low.

Finally, let us discuss whether the outcome of the inelastic events, i.e. structural damage versus relaxation, is stochastic. As seen in Figs. 2(a)–(d), it is evident that the inelastic events can cause either structural damage (p-type spike) or relaxation (n-type spike). While this finding contradicts the original STZ model^32^, which assumed that the local inelastic events are the source of structural disordering (damage), however, it supports the two-state STZ model later developed by Falk and Langer^10^, in which it was postulated that a local inelastic event has two opposite effects: it can cause either jamming (relaxation) or disordering (damage). Furthermore, we have shown that the magnitude of these local inelastic events follows a power-law distribution conforming to the behavior of self-organized or tuned criticality^7^,^36^,^37^. However, it needs to point out the outcome of these inelastic event is not ‘stochastic' at all, which clearly shows the stress dependence and also the stress-rate dependence. To avoid the complexity in counting the magnitude of the spike event, Figure 5 only displays the difference between the numbers of the p- and n-type spikes occurring under different stress conditions. In doing so, we are only interested to know the relative frequency of the two types of events. Interestingly, it can be seen that more p-type spikes tend to occur during loading, signifying the damaging of the amorphous structure, while more n-type spikes tend to occur during unloading, signifying the recovery of the amorphous structure. More interestingly, the numbers of the p- and n-type spikes tend to be equal at load hold, indicative of the situation of a dynamic equilibrium. These findings indicate that, through the competition of the different local inelastic events, the metallic glass can sustain local damage as well as heal itself through structural relaxation. To our knowledge, this is the first experimental report of this important ‘self-healing' mechanism as mediated through the activation of local inelastic events, which has many important implications and is worth further research.

To conclude, it is demonstrated in this Letter that, with the state-of-art NanoECR technique, the local inelastic events hidden, otherwise, in the overall elastic response of the metallic glass could be revealed. Due to the elastic coupling, these local inelastic events take place cooperatively, exhibiting a self-organized behavior as seen in their magnitude distribution. In contrast to the classic model, it is also shown that the effects of these inelastic events are twofold: it can either damage or heal the amorphous structure. Depending on the applied stress and stress rate, the competition of the local events can lead to overall damage or recovery of the amorphous structure. These intriguing and multi-scaled deformation characters as seen in the metallic glass are parallel to those already known phenomena observed for earthquakes, sand pile avalanches, dislocation slip dynamics and a variety of other dynamic systems^36^,^38^, implying the universality of the underlying deformation dynamics across the general field of materials science and condensed matter physics.

## Methods

The Zr-based metallic-glass micropillars were prepared by FEI Quanta 3D 200 FIB/SEM dual beam system. Following the ion-milling method detailed in Ref.^17^, the constant voltage of 30 keV, the current density of Gallium ion beams ranging from 7 nA to 100 pA was used to carve out the micropillars. The micropillars have the top diameters ranging from 1.5 ~ 2.2 μm and the aspect ratios from 2.1–2.8. Same as the literature results, the taper angle is well controlled below 3°. The nanoECR tests were subsequently conducted on the micropillars with load control using a 10-μm flat-end diamond punch. The loading, holding and unloading times were all fixed at 10 seconds and a constant voltage of +5 V was applied throughout the whole microcompression tests. The peak load was started from 500 μN and then increased to 1000 μN, 1500 μN, 2000 μN and all the way till 6000 μN with same testing parameters. Below 6000 μN, no obvious displacement bursts could be observed. To correlate the pop-in strain and the resistivity spike, the load was further increased to 7000 μN to trigger prominent pop-in events. The similar experiments were repeated on other micropillars to ensure data repeatability.

## Author Contributions

C.T.L. and Y.Y. designed the project. S.W. and Z.Y.L. carried out the experiments. X.L.F., B.A.S., Y.F.Y., Y.Y. and C.T.L. analyzed the data. Y.Y. and X.L.F. wrote the manuscript.

## Supplementary Material

Supplementary Information Supplementary Information

## Figures and Tables

**Figure 1:**
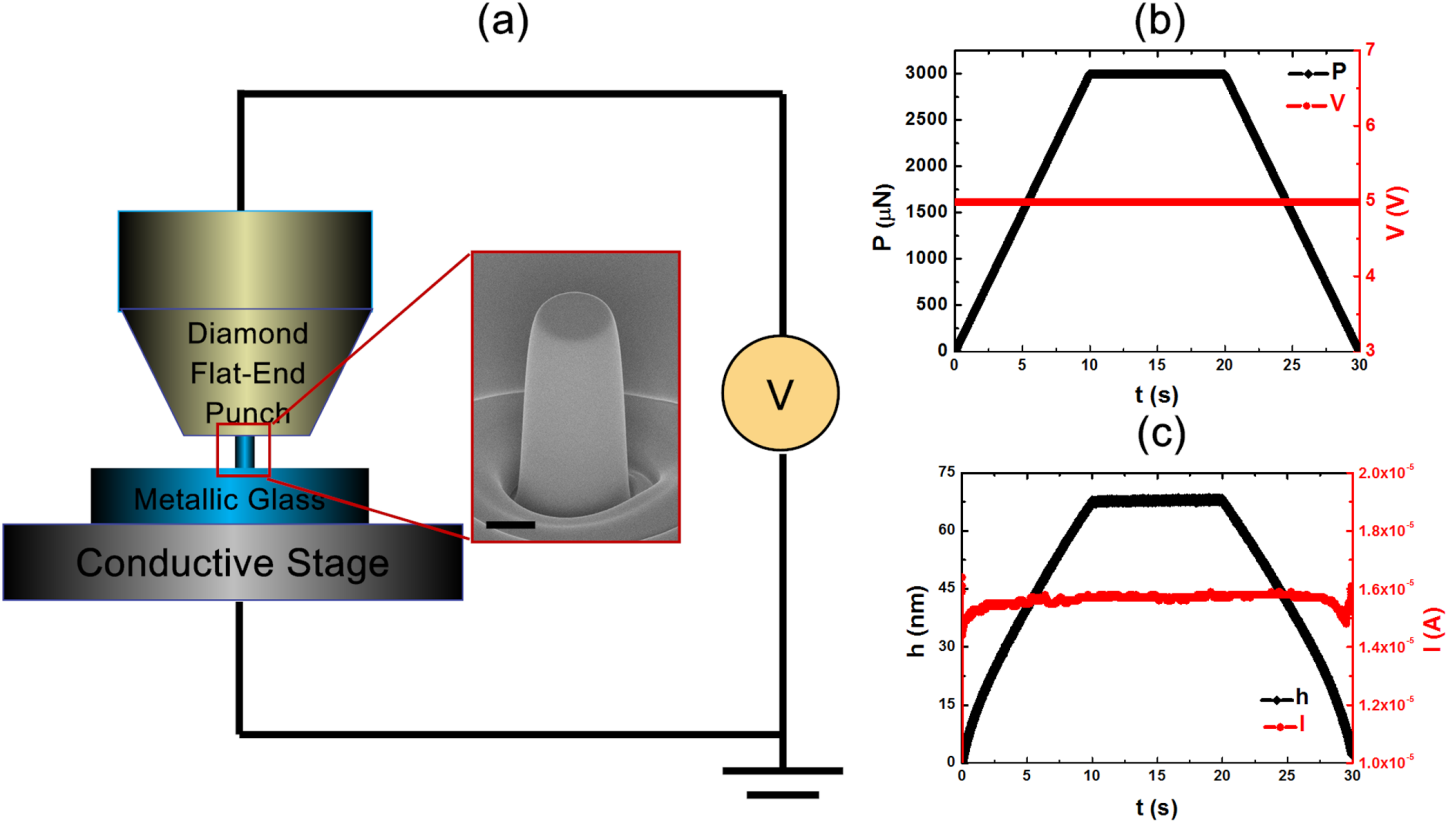
The experimental set-up of and typical data obtained directly from the NanoECR-coupled microcompression test. (a) The schematics showing the set-up of the microcompression test [Note that the drawing is not to scale and the inset shows the scanning electron microscopy (SEM) image of a Zr-based metallic-glass micropillar (scale bar = 1 μm), (b) the constant voltage (V) superimposed on the load (P) function consisting of the loading, holding and unloading segments, and (c) the measured displacement (*h*) of and the electric current (*I*) passing through the deformed micropillar.

**Figure 2:**
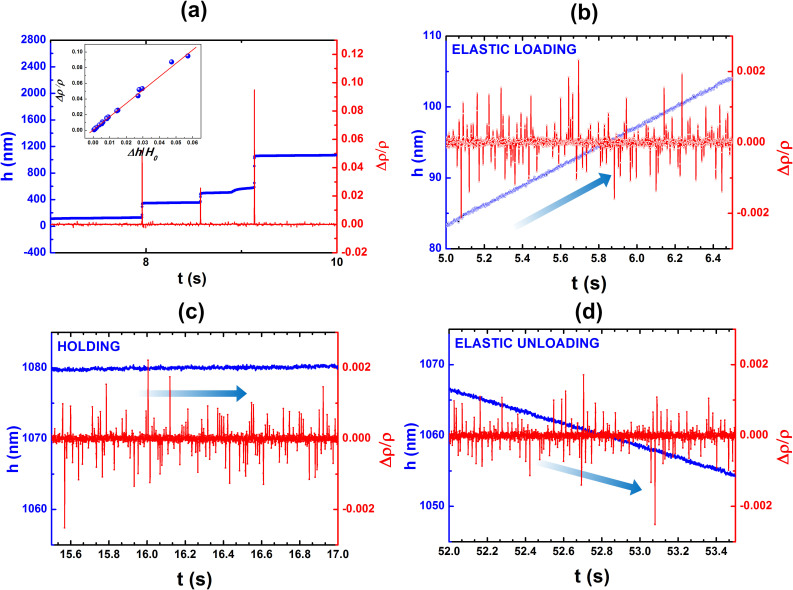
The experimental data revealing the deformation-induced inelastic events in the metallic-glass micropillars. (a) The variations of the displacement (*h*) and the relative change in the electric resistivity (*Δρ/ρ*) with time (*t*) (displacement = blue circle and electric resistivity = red line) [Inset: the correlation between *Δρ/ρ* measured at pop-in and the corresponding strain jump *Δh/H_0_* (*H_0_* = the original height of the micropillar]; the magnified views of the *h* and *Δρ/ρ* versus *t* curves obtained during (b) elastic loading, (c) holding, and (d) unloading.

**Figure 3:**
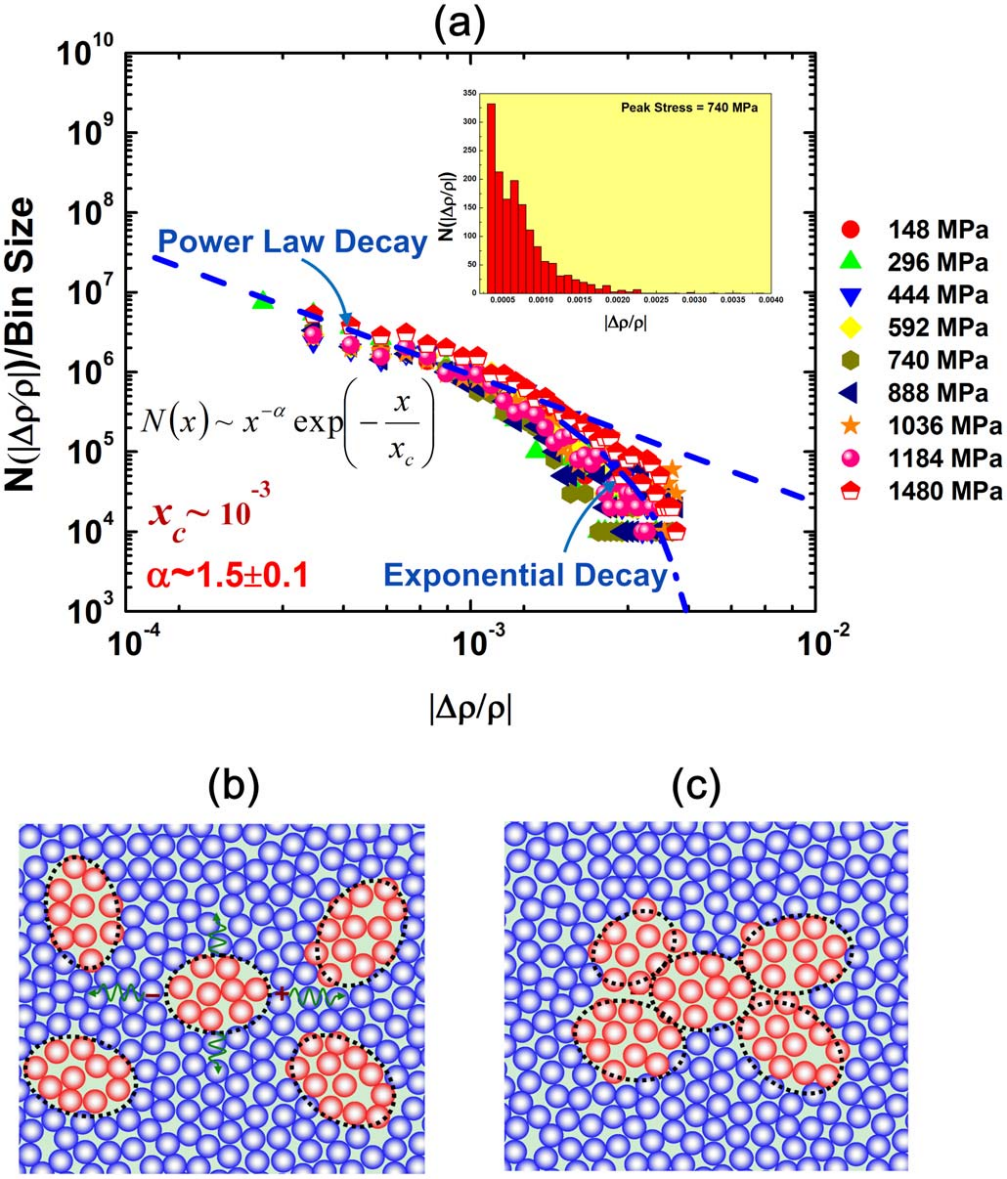
The stochastic behavior of the secondary spikes arising before yielding occurs in the metallic-glass micropillar. (a) The distribution of the event magnitude for various peak stresses (inset: The histogram of the spike magnitude obtained when the stress increases from 0 to a maximum stress of 740 MPa), the schematics showing the possible mechanisms of cooperative shear transformations due to (b) elastic stress coupling and (c) de-caging or coalescence of local liquid-like zones. (red circles = atoms in the liquid-like zone and blue circles = atoms in the elastic matrix).

**Figure 4:**
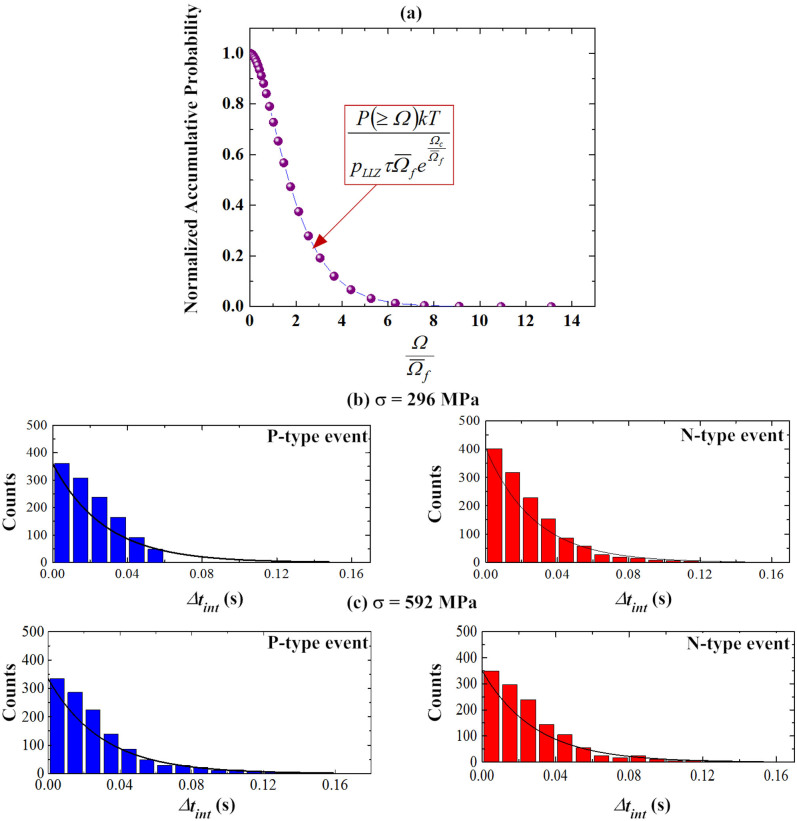
(a) The curve of the normalized accumulative probability versus the normalized activation volume; and the distributions of waiting times between consecutive like events for the holding stresses of (a) 296 MPa and (b) 592 MPa.

**Figure 5:**
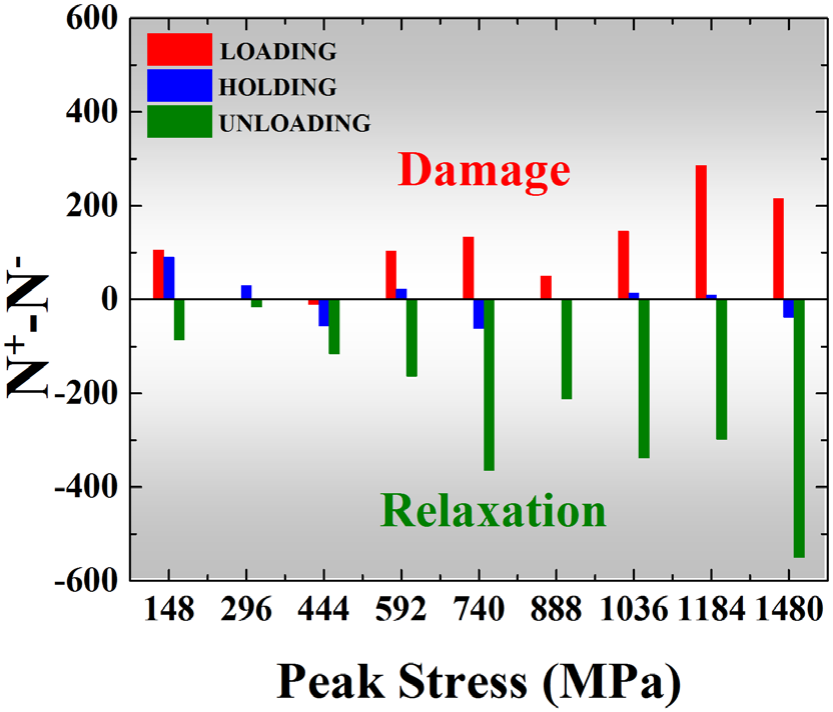
The effects of damage versus relaxation induced by local inelastic events. The bar charts showing the difference between the numbers of the p-type spikes (N^+^) and the n-type spikes (N^−^) occurring under different stress conditions (Note that the more detailed discussion on the competition between the two types of spikes is seen in the Supplementary Information).
