# Supplementary material for: Probing Stochastic Nano-Scale Inelastic Events in Stressed Amorphous Metal
Source: Sci Rep. 2014 Oct 21;4:6699. doi: 10.1038/srep06699 (PMC4204032; doi:10.1038/srep06699)
Supplement: Supplementary Information [file srep06699-s1.pdf]

Supplementary Information

Probing Stochastic Nano-Scale Inelastic Events in Stressed Amorphous Metal

Y. Yang*, X.L. Fu, S. Wang, Z.Y. Liu, Y.F. Ye, B.A. Sun, C.T. Liu

Centre for advanced structural materials, Department of Mechanical and Biomedical Engineering, City University of Hong Kong, Tat Chee Avenue, Kowloon Tong, Kowloon, Hong Kong, P.R. China

1. Calibration of NanoECR

The calibration was started on an open circuit by coating an insulating ultra-thin layer of wax on the indentation tip. This tip was subsequently pressed slightly onto the micropillar for detecting the noise level. The electrical signals so detected were shown in Fig. S1. The red line shows the noise level of ±100 pA for the current measured at the fixed voltage of 5V (the blue line), According to this calibration, the current resolution of ±100 pA and the voltage resolution of ±0.02 mV are obtained.

2. Structural Characterization

The model metallic glass has a chemical composition of $Zr_{55}Cu_{30}Ni_{5}Al_{10}$ (in atomic %), which has already been studied in our previous work[1]. Its XRD pattern and high-resolution transmission electron microscopy image are shown in Figs. S2 and S3(a)-(b), which confirms the structural amorphousness.

3. Electric Contact Analysis in Microcompression

As described in the method, the whole testing time is 30s. The displacement and

* To whom correspondence should be addressed. Email: yonyang@cityu.edu.hk

electric data can only be legitimate after the indenter and micropillar are in full contact. As shown in Fig. S1 or Fig. 1c, a nonlinear current-voltage behavior can be noticed at the initial stage when the nanoindenter is engaged with the micropillar, or at the final stage when these two are disengaged. According to Refs[2-4], such a nonlinear feature results from the nano-scale electrical contact, which can be attributed to the settling of the nanoindenter on the micropillar. At this stage, due to the constricted flow of electrons passing through the nano-contact between the indenter and the asperities on the micropillar surface, the material's electric resistance appears significantly higher than what it should be at an Ohm's contact. To avoid complexity, we discarded all the data collected within the settling periods. Normally it took less than 2 seconds for the indenter to settle. However, for a conservative analysis, we took out the data collected in the first 5 seconds during loading and in the last 5 seconds during unloading.

3. Ohm's Law in Microcompression

Considering a thin disk with an infinitesimal height dz, the net electric current dI flowing through it at the constant voltage V can be expressed as follows

$$dI = \frac{Vdz}{\rho A(z)} \tag{S1}$$

where ρ denotes the electric resistivity and $A(z)$ the cross sectional area of that disk (Fig. S4). Given $A(z) = A_0\left(1 + \frac{z}{r_0}\tan\beta\right)^2$ where β is the taper angle; r_0 is the radius of the top area of the micropillar and $A_0 = \pi r_0^2$. Integrating (S1) from $z = 0$ to $z = H$ gives the electric current I flowing through the whole micropillar:

$$I=\frac{VH}{\rho A_0\left(1+H/r_0\tan\beta\right)}=\alpha\left(\frac{H}{r_0},\tan\beta\right)\frac{VH}{\rho A_0} \tag{S2}$$

where $\alpha\left(\frac{H}{r_0},\tan\beta\right)=\frac{1}{\left(1+H/r_0\tan\beta\right)}$. Taking the natural logarithm of (S2) and performing the differentiation, we obtain

$$\frac{dI}{I}=\frac{d\alpha}{\alpha}+\frac{dH}{H}-\frac{d\rho}{\rho}-\frac{dA_0}{A_0} \tag{S3}$$

in which:

$$\frac{dH}{H}=d\varepsilon_T \tag{S4}$$

$$\frac{dA_0}{A_0}=\frac{2dr_0}{r_0}=-2vd\varepsilon_T \tag{S5}$$

$$\frac{d\alpha}{\alpha}=-\frac{\tan\beta d\left(\frac{H}{r_0}\right)+\frac{H}{r_0}d\left(\tan\beta\right)}{\left(1+\frac{H}{r_0}\tan\beta\right)} \tag{S6}$$

where $d\varepsilon_T$ is the infinitesimal true strain and v the Poisson's ratio.

In general, it can be easily derived that

$$d\left(\frac{H}{r_0}\right)=\left(\frac{H}{r_0}\right)\frac{dH}{H}-\left(\frac{H}{r_0}\right)\frac{dr_0}{r_0}=\left(1+v\right)\left(\frac{H}{r_0}\right)\frac{dH}{H}=\left(1+v\right)\left(\frac{H}{r_0}\right)d\varepsilon_T \tag{S7}$$

Now let us discuss the differential change in the taper angle. As shown in Fig. S4, $\tan\beta=(r_b-r_0)/H$, where r_b is the base radius. Assuming that r_b remains constant during the elastic deformation, we have:

$$d\left(\tan\beta\right)=\frac{-Hdr_0-\left(r_b-r_0\right)dH}{H^2}=-\left(\frac{r_0}{H}\right)\frac{dr_0}{r_0}-\tan\beta\frac{dH}{H}=\left(v\frac{r_0}{H}-\tan\beta\right)d\varepsilon_T \tag{S8}$$

Inserting (S8) and (S7) into (S6) then gives:

$$\frac{d\alpha}{\alpha}=vd\varepsilon_T \tag{S9}$$

Combining (S3), (S4), (S5) and (S9), we have

$$\frac{d\rho}{\rho} = (1+3\nu)d\varepsilon_T - \frac{dI}{I} \qquad \text{(S10)}$$

Since the Poisson's ratio ν roughly equals 1/3 for metals and $\varepsilon_T = ln(1+\varepsilon)$ where ε denotes the engineering strain, we finally obtain

$$\frac{d\rho}{\rho} = \frac{2d\varepsilon}{1+\varepsilon} - \frac{dI}{I} \qquad \text{(S11)}$$

Note that (S11) is the exact solution obtained after considering both the size and geometric change in the deformed tapered micropillar. However, as discussed below, the value of $d\rho/\rho$ is dominated by the relative change in the electric signal dI/I rather than the mechanical signal $d\varepsilon/(1+\varepsilon)$.

4. Error Analysis

Before extracting the spikes using Eq. (S11), it is critical to differentiate the real structural signal from the system noise. According to Eq. (S11), the noise-to-signal ratio for the derived term $\Delta\rho/\rho$ is a combination of those for the two measurements $2\Delta\varepsilon/(1+\varepsilon)$ and $\Delta I/I$. The noise-to-signal ratio for $\Delta I/I$ can be simply calculated as the current resolution 100 pA divided by the measured current 10 μA, which is about 1×10^{-5}. On the other hand, since $2\Delta\varepsilon/(1+\varepsilon) \sim 2\Delta\varepsilon$, the noise-to-signal ratio for the term $2\Delta\varepsilon/(1+\varepsilon)$ can be approximated as that for $2\Delta\varepsilon$, which equals to two times of the displacement resolution ~0.2 nm divided by the average micropillar height ~4 μm, which is about 5×10^{-5}. Therefore, we estimate that the noise-to-signal ratio for the derived $\Delta\rho/\rho$ should be on the order of 1×10^{-4}.

To confirm our theoretical estimation, the curves of $\Delta\rho/\rho$ and $2\Delta\varepsilon/(1+\varepsilon)$ versus t are both plotted in Fig. S5(a). From Fig. S5(a) and the inset, it is evident that the

general trends shown by the two curves are not similar. The extracted Δρ/ρ data ranges from 1×10^{-3} to 0.1, at least one order of magnitude larger than the corresponding values of 2Δε/(1+ε), indicating that the change in the electric resistivity Δρ/ρ is dominated by that in the current *ΔI/I*, while 2Δε/(1+ε) only contributes to a very small portion of Δρ/ρ. Furthermore, the distribution of 2Δε/(1+ε) is shown in Fig. S5(b), which can be well fitted by a Gaussian curve. If the standard deviation of the Gaussian fit is taken as the experimental error, the noise-to-signal ratio for 2Δε/(1+ε) can be thus approximated as 1×10^{-4}, which agrees with the theoretical estimation.

5. Ziman's Model

According to Ziman's theory[5], the electrical resistivity ρ for pure liquid metals can be expressed as

$$\rho = \frac{3\pi\upsilon}{4\hbar e^2 v_F^2 k_F^4} \int_0^{2k_F} S(q)|\mu(q)|^2 q^3 dq \tag{S12}$$

where k_F is the Fermi wave vector, v_F is the Fermi velocity, S(q) is the structure factor and μ(q) is the pseudo-potential defined in the Ziman's model.

According to Kelton and Spapen[6], the change of resistivity can be expressed as

$$\frac{\Delta\rho}{\rho_0} = \frac{(\partial\rho/\partial\xi)_{\xi(0)}}{\rho_0}\Delta\xi \tag{S13}$$

$$\frac{(\partial\rho/\partial\xi)_{\xi(0)}}{\rho_0} \equiv K = \frac{k_F^0}{3\xi(0)}\left[p - (9/k_F^0) - 2r\right] \tag{S14}$$

where

$$p = \frac{\int_0^{2k_F^0} [\partial S(q,\xi)/\partial\xi]_{\xi(0)} q^3 |\mu(q)|^2 dq}{\int_0^{2k_F^0} S[q,\xi(0)] q^3 |\mu(q)|^2 dq} \frac{3\xi(0)}{k_F^0} \tag{S15}$$

$$r = \frac{S\left[2k_F^0, \xi(0)\right](2k_F^0)^3\left|u(2k_F^0)\right|^2}{\int_0^{2k_F^0} S\left[q, \xi(0)\right]q^3\left|\mu(q)\right|^2 dq} \tag{S16}$$

In Eq. S13, $\Delta\xi = \xi(t+\Delta t) - \xi(t)$, $\xi(t)$ is the as-quenched atom packing fraction at time t and $\Delta\xi$ represents the change of the packing fraction across a time period of Δt. Here, the preterm $\frac{(\partial\rho / \partial\xi)_{\xi(0)}}{\rho_0}$ can be regarded as a constant since the structural factor of metallic glasses is nearly not affected by thermal annealing and mechanical stress.

6. The Spike Lasting Time

The above error analysis assures that our NanoECR system with a noise-to-signal ratio of 1×10^{-4} is capable of detecting any 'tiny' change occuring in $\Delta\rho/\rho$. For a conservative analysis, a threshold of 3×10^{-4} was selected to single out the $\Delta\rho/\rho$ data from the system noise. For a systematic investigation, we also tried other thresholds, such as 5×10^{-4} and 8×10^{-4}, and found that the results were not sensitive to the thresholds chosen from the selected range.

After choosing the threshold of $\pm3\times10^{-4}$, a Matlab™ based program was developed to single out the different spikes, including both p- and n-type occurring during the 'elastic' regime. Afterwards, the nominal lasting time Δt_j of the jth spike could be estimated as the difference between the time points $\Delta t_j = t_{j(end)} - t_{j(start)}$ when the data point enters and exits from the spike. To avoid ambiguity in tracing the spike tails, the spike lasting time Δt was finally chosen as that corresponding to the full width at half maximum (HWFM) of the spike, which roughly equals $\Delta t_j/2$. Due to the temporal resolution of our system, the lasting times for the secondary spikes are

distributed within a narrow range centered at around 1.5 ms, irrespective of the spike type and the external stress condition (Fig. S6).

7. Estimation of Power law Exponent

Following the method in Ref.[7,8]. the power-law exponent α in Fig. 3(a) can not only be obtained through data fitting but also be estimated using the following equation,.

$$\alpha = 1 + n\left[\sum_{i=1}^{n} \ln \frac{x_i}{x_{\min}}\right]^{-1} \tag{S17}$$

Here the quantities x_i, i=1, 2, …n are the measured values of x and x_{min} is the minimum value of x. Given $|\Delta\rho/\rho|_{min} \sim 1\times10^{-4}$, α is estimated as 1.5. Meanwhile, the statistical error σ for α is:

$$\sigma = \sqrt{n}\left[\sum_{i=1}^{n} \ln \frac{x_i}{x_{\min}}\right]^{-1} = \frac{\alpha - 1}{\sqrt{n}} \tag{S18}$$

which gives σ = 0.1 at α =1.5.

8. The Estimation of Activation Energy

Although the local inelastic events occur cooperatively, we can also apply the mean-field modeling to estimate their activation energy by treating the elastically coupled local events as one subcritical event. According to the stochastic shear transformation model[9], the constitutive equation for this subcritical event is:

$$\frac{d\gamma}{dt} + 2\omega e^{-\frac{\Delta G}{kT}}\gamma = 2\left(\frac{\beta\Omega}{kT} + \frac{1}{\mu}\right)\omega e^{-\frac{\Delta G}{kT}}\tau \tag{S19}$$

where τ denotes the applied shear stress; γ denotes the total strain; ω and ΔG denote the attempt frequency and the energy barrier against a local configuration transition, respectively; t is time, k the Boltzmann constant, T the ambient temperature and Ω the activation volume for a local configurational transition event.

Note that Eq. (S19) is essentially a rheological relation, from which the event lasting time t_R can be derived as follows:

$$t_R = \frac{1}{2\omega} exp\left(\frac{\Delta G}{kT}\right) \tag{S20}$$

Inserting $\omega=10^{13}$ s^{-1} (the Debye frequency), t_R ~ 1-1.5 ms, and T = 300 K into (S20), one can estimate the activation energy ΔG of ~0.5 eV for the subcritical event at room temperature, which is on the same order of magnitude of the previous estimations reported in the literature[10].

References

1 Ye, J. C., Lu, J., Liu, C. T., Wang, Q. & Yang, Y. Atomistic free-volume zones and inelastic deformation of metallic glasses. *Nat. Mater.* **9**, 619-623, (2010).

2 Stauffer, D. D. *et al.* Plastic response of the native oxide on Cr and Al thin films from in situ conductive nanoindentation. *J. Mater. Res.* **27**, 685-693, (2012).

3 Tonck, A., Houze, F., Boyer, L., Loubet, J. L. & Georges, J. M. ELECTRICAL AND MECHANICAL CONTACT BETWEEN ROUGH GOLD SURFACES IN AIR. *Journal Of Physics-condensed Matter* **3**, 5195-5201, (1991).

4 Kim, D. I., Pradeep, N., DelRio, F. W. & Cook, R. F. Mechanical and electrical coupling at metal-insulator-metal nanoscale contacts. *Appl. Phys. Lett.* **93**, (2008).

5 Ziman, J. M. A theory of the electrical properties of liquid metals. I: The monovalent metals. *Phil. Mag.* **6**, 1013-1034, (1961).

6 Kelton, K. F. & Spaepen, F. KINETICS OF STRUCTURAL RELAXATION

IN SEVERAL METALLIC GLASSES OBSERVED BY CHANGES IN ELECTRICAL-RESISTIVITY. *Phys. Rev. B* **30**, 5516-5524, (1984).

7 Yang, Y. *et al.* Fractal growth of the dense-packing phase in annealed metallic glass imaged by high-resolution atomic force microscopy. *Acta Materialia* **60**, 5260-5272, (2012).

8 Dimiduk, D. M., Woodward, C., LeSar, R. & Uchic, M. D. Scale-free intermittent flow in crystal plasticity. *Science* **312**, 1188-1190, (2006).

9 Huo, L. S., Zeng, J. F., Wang, W. H., Liu, C. T. & Yang, Y. The dependence of shear modulus on dynamic relaxation and evolution of local structural heterogeneity in a metallic glass. *Acta Mater.* **61**, 4329-4338, (2013).

10 Greer, A. L., Cheng, Y. Q. & Ma, E. Shear bands in metallic glasses. *Materials Science and Engineering: R: Reports* **74**, 71-132, (2013).

List of Figure Captions

Figure S1. The X-ray diffraction pattern of the as-cast Zr-based metallic glass showing the overall structural amorphousness.

Figure S2. The high resolution transmission electron microscopy (TEM) image of the as-cast $Zr_{55}Cu_{30}Ni_5Al_{10}$ bulk metallic glass showing a typical maze-like pattern; and (b) the Fast-Fourier-Transformation (FFT) filtered image of (a) confirming the amorphous atomic structure of the as-cast $Zr_{55}Cu_{30}Ni_5Al_{10}$.

Figure S3. The current- and voltage-time curves obtained from the NanoECR technique when the system is open-circuited by applying a thin layer of wax on the flat-end punch. It shows that the absolute voltage resolution can reach about ±0.02 mV while the current resolution about ±100pA at the applied voltage of +5V.

Figure S4. The schematics showing the geometry and size change in the micropillar after elastic deformation.

Figure S5. (a) Comparison between the variations of $\Delta\rho/\rho$ and $2\Delta\varepsilon/(1+\varepsilon)$ with the time t. Note the sudden change in $\Delta\rho/\rho$ due to the indentation settling (inset: the close-up view of the two curves) ; and (b) The distribution of the measured $2\Delta\varepsilon/(1+\varepsilon)$ that can be fitted perfectly with the Gaussian curve with the standard deviation at 1.3×10^{-4}.

Figure S6. The power-law like distributions of the spike lasting times measured during loading, holding and unloading (the peak stress = 740 MPa).

List of Figures

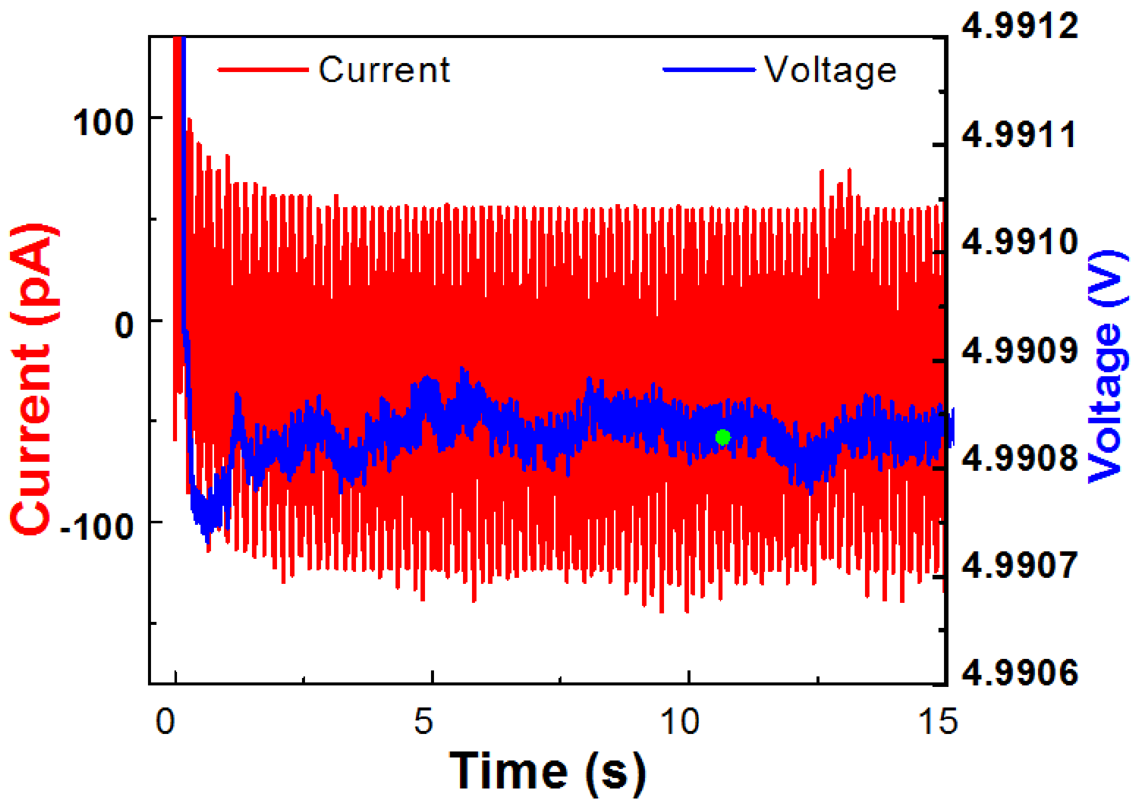

Fig. S1. Yang *et al*.

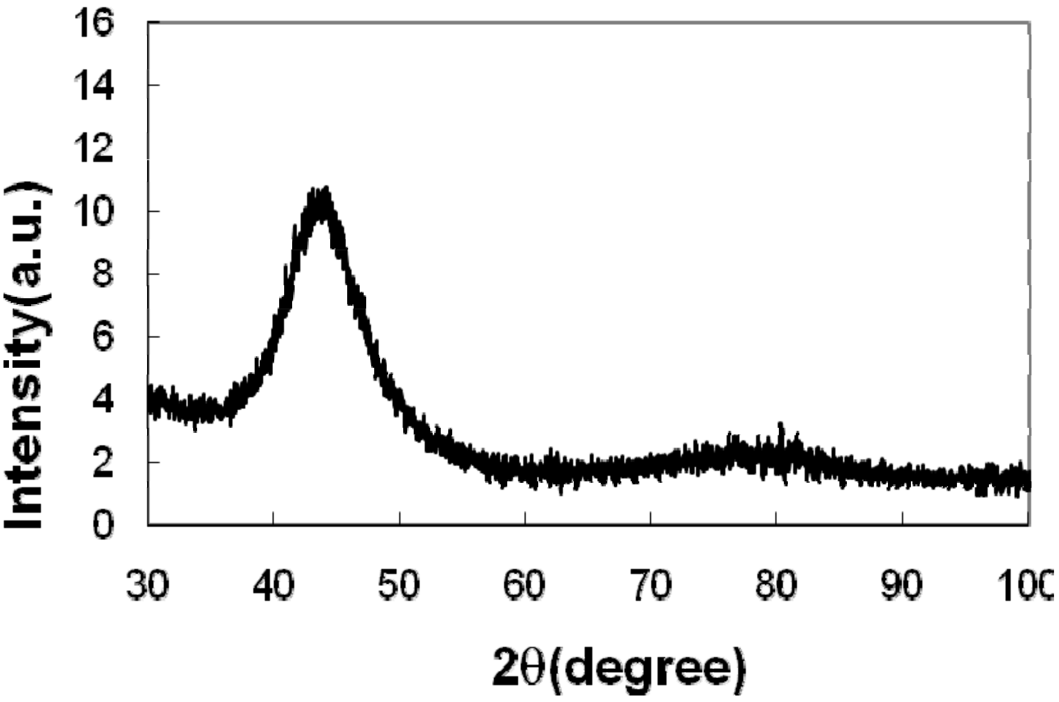

Fig. S2. Yang *et al*.

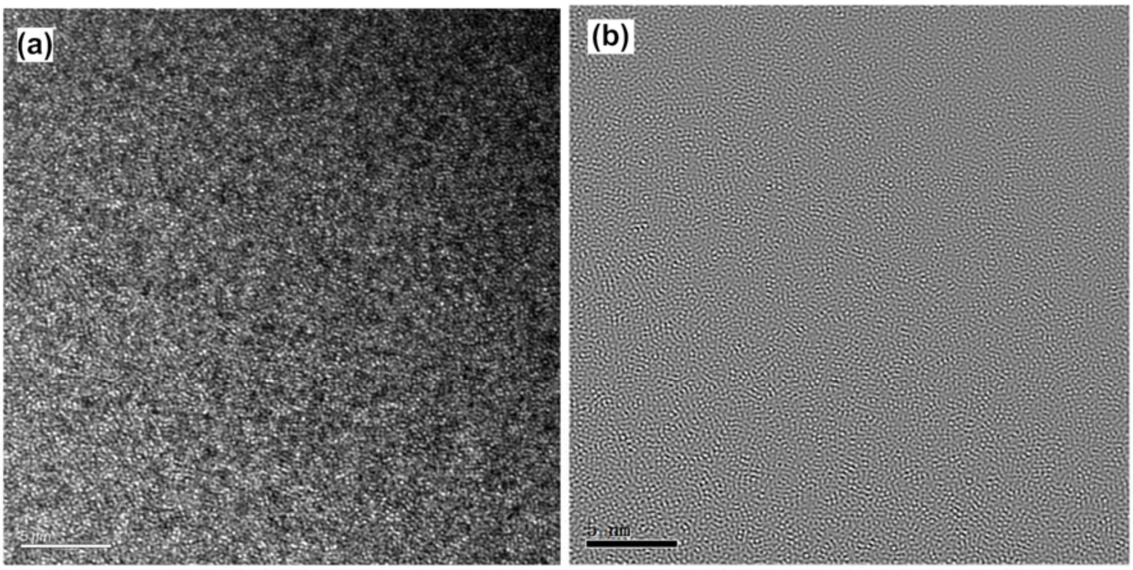

Fig. S3. Yang *et al*.

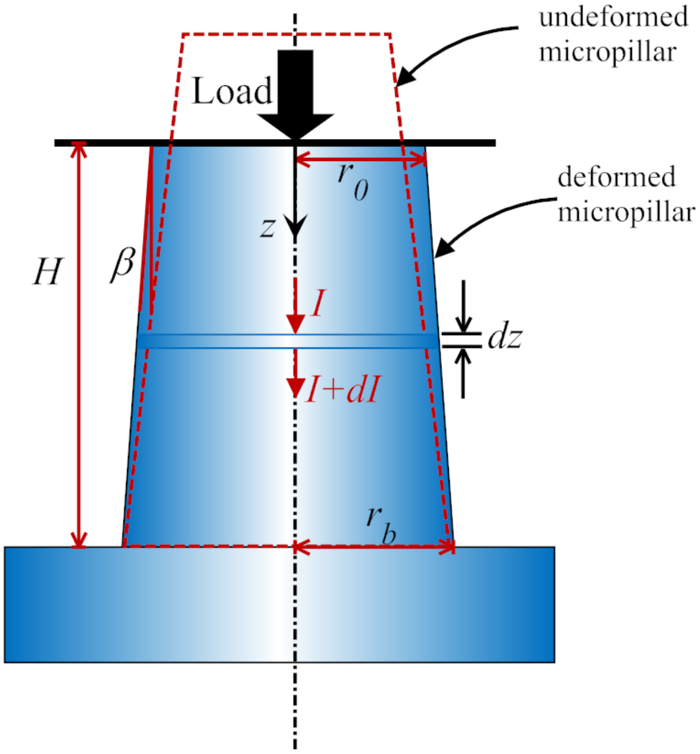

Figure S4. Yang *et al.*

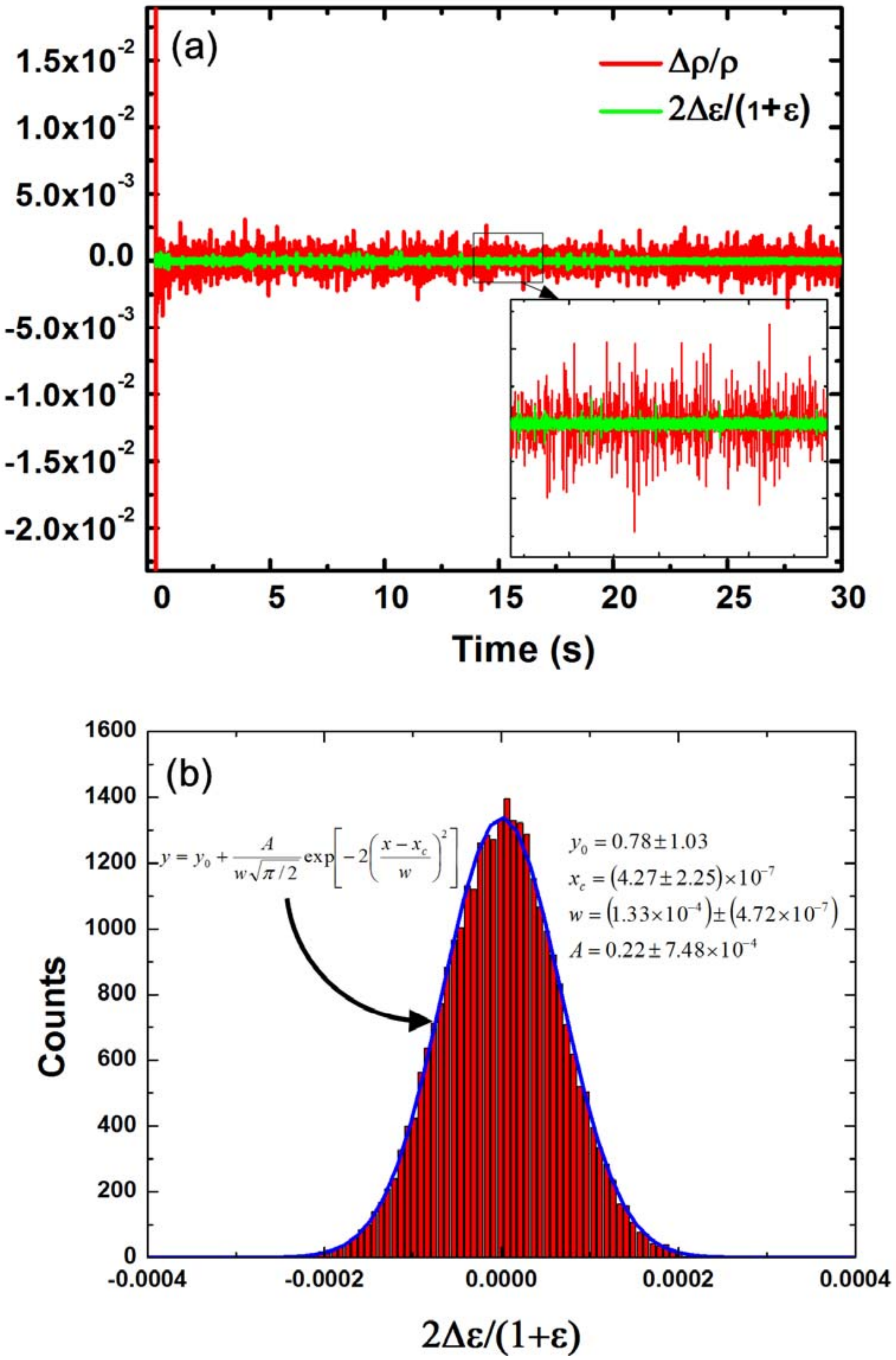

Fig. S5. Yang *et al*.

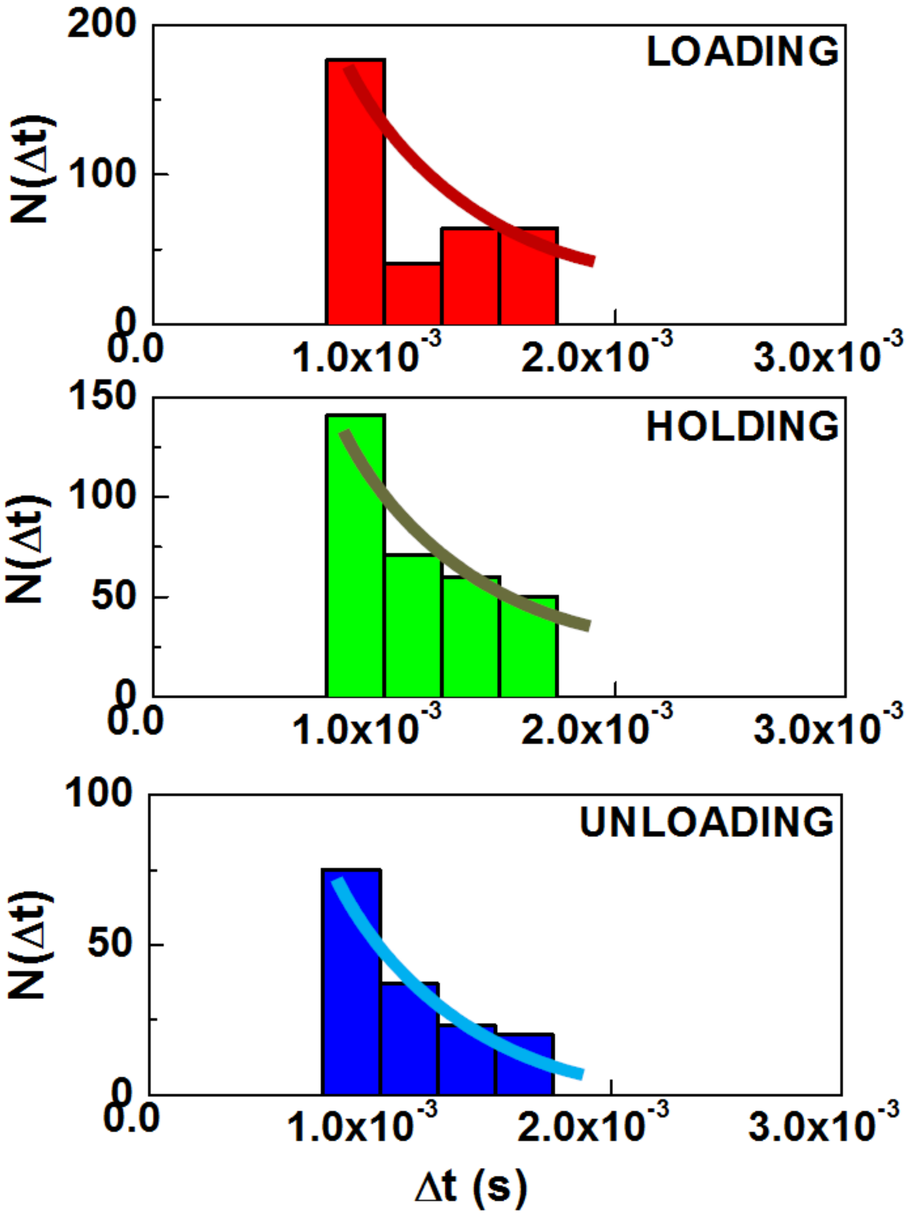

Fig. S6. Yang *et al*.
